# Approach–Avoidance pattern of attentional bias in individuals with high tendencies toward problematic Internet pornography use

**DOI:** 10.3389/fpsyt.2022.988435

**Published:** 2022-09-08

**Authors:** Jianfeng Wang, Yalian Huang

**Affiliations:** School of Psychology, Chengdu Medical College, Chengdu, China

**Keywords:** problematic Internet pornography use, attentional bias, approach–avoidance pattern, ambivalence, behavioral addiction

## Abstract

Attentional bias plays a vital role in the occurrence and development of addictive behaviors. However, little is known about attentional processes in problematic Internet pornography use (PIPU), and previous studies have reported mixed results. The current study examined the components of attentional processing to sexual stimuli using an exogenous cueing task designed to differentiate between attentional engagement and disengagement. Two different stimulus presentation times (100 and 500 ms) were used to present the pornographic and neutral images to differentiate the early and late stages of attentional bias. Individuals with high (*n* = 40) and low (*n* = 40) PIPU tendencies were compared. The results demonstrated that individuals with high tendencies toward PIPU showed enhanced attentional engagement with pornographic stimuli in the early stage of attentional processing (100 ms), followed by attentional avoidance in the late stages of attentional processing (500 ms). Moreover, the severity of PIPU symptoms was positively correlated with attentional engagement scores in the short picture-time trials (100 ms) and weakly negatively correlated with attentional disengagement scores in the long picture-time trials (500 ms). This approach–avoidance pattern of attentional biases is in line with a recent theoretical model that emphasizes that appetitive and aversive motivational processes jointly determine attentional bias.

## Introduction

In the past few decades, the consumption of online pornography has increased exponentially (1). An online survey estimated the lifetime prevalence of pornography viewing to be approximately 92–98% for men and 50–91% for women (2). Although pornography viewing is a form of entertainment for most people, some individuals develop subjective symptoms and negative consequences as a result of problematic participation in this activity (3, 4). This problematic Internet pornography use (PIPU) is defined as the inability to control the excessive use of Internet pornography, despite its serious negative outcomes (4).

Although the classification of PIPU remains controversial, several scholars believe that it should be conceptualized as behavioral addiction, which contains the key ingredients for addiction, such as withdrawal, tolerance, loss of control, and compulsive use (5–8). There is growing evidence that PIPU resembles substance use disorder (9, 10). For instance, previous studies have identified cue reactivity and craving as an important mechanism of PIPU (11–13) equally important for the development and maintenance of substance dependence (14). In addition, the neural processing of sexual- and substance-related cues relies on identical brain networks, possibly regulated by the mesolimbic dopamine system (15, 16). Furthermore, the dual-process model of addiction (17) argues that addictive behaviors are influenced by competing impulsive and reflective systems. However, in addictive individuals, the reflective system is overridden by the impulsive system. Similarly, individuals with PIPU exhibit impaired response inhibition during processing of sexual stimuli (18, 19).

The current cognitive model of addiction suggests that enhanced attentional capture of addiction-related cues plays a vital role in the persistence of addictive behaviors (20). That is, addicted individuals tend to automatically and uncontrollably allocate attentional resources to specific types of stimuli associated with the individual's current concern (21, 22). According to incentive-sensitization theory (22, 23), chronic exposure to addictive stimuli alters the brain's reward circuits (particularly the mesolimbic dopamine system), resulting in neural sensitization that increases the motivational appeal of the addictive stimuli. Using classical conditioning, addiction-related cues obtain incentive salience and thus capture attention and form attentional biases.

According to Posner's attentional processing model (24), three types of bias may contribute to PIPU. First, individuals with PIPU may exhibit attentional vigilance toward sexual cues, prompting them to detect sexually relevant stimuli in the environment. The amygdala has been implicated as a neural mechanism that may mediate automatic vigilance (25). This hyper vigilance leads to enhanced cognitive processing of sexual stimuli, which may further contribute to cravings for these stimuli. Second, individuals with PIPU may have difficulty disengaging from sexual stimuli. Prefrontal regulatory structures may underlie delayed disengagement via individual differences in the attentional control ability (26). Maintaining attention to these stimuli can cause individuals to experience chronic cravings, which can lead to persistence and maintenance of the disorder. Third, individuals with PIPU may exhibit attentional avoidance, which is the tendency to avoid specific cues and divert attention away from them. Emotion regulation goals may mediate attentional avoidance, a process that may be neurally centered around prefrontal cortex activity (26). Evidence from substance addiction and obesity studies suggests that attentional avoidance of addictive stimuli may be a strategic way to regulate negative emotional responses and suppress subjective cravings [see (27) for a review]. Similarly, individuals with PIPU may show attentional avoidance of sexual stimuli in an attempt to escape the negative emotions that arise from excessive viewing of Internet pornography.

To date, few studies have investigated attention-related cognitive processing in PIPU. One of the most commonly used methods to assess attentional bias is the Stroop task. In this task, participants are required to identify the color of the words while ignoring their semantic content. It is assumed that slower color naming on trials with the addiction-related words indicates attentional processing of the meaning of the words, which interferes with color naming. Using the modified addiction Stroop task (21), individuals who are sexually active have been found to respond more slowly to sexually related words than to neutral words (28). Using the same task, but with picture stimuli, Wang et al. (13) found that individuals with cybersex addiction exhibited a Stroop interference effect for pornographic images relative to neutral images. Another commonly used attentional instrument is the visual dot-probe task. In this task, participants are presented with two stimuli, one addiction-related and one neutral, at two different spatial locations on the screen. After these stimuli disappeared, a probe appears in the locations just occupied by one of them. Participants are instructed to respond to the probe as rapidly as possible. It stands to reason that the response to the probe will be faster if the attention is already allocated to location where the probe appears. Using this instrument, Mechelmans et al. (29) found that individuals with compulsive sexual behavior disorder (CSBD; PIPU is the main symptom) exhibited enhanced attentional bias to sexually explicit rather than neutral pictures when the stimulus presentation time was 150 ms. However, Doornwaard et al. (30) found that when the stimulus presentation time was extended to 500 ms, moderate and high pornography users showed no attentional bias toward sexual stimuli relative to low pornography users. Furthermore, attentional processing of addiction-related stimuli can be assessed using event-related potentials (ERPs). Wang et al. (13) compared the ERPs activity of participants with high and low cybersex addiction while performing an addiction-Stroop task. They found larger P200 (150–220 ms) amplitudes for sexual images than neutral images in the high cybersex addiction group. However, for late ERP components, the high cybersex addiction group elicited lower late positive potential (LPP) amplitudes for both pornographic and neutral stimuli than the low cybersex addiction group (13). This finding replicates previous ERP studies that also observed lower LPP amplitudes for sexual images in frequent porn viewers than in healthy controls (31). These electrophysiological results demonstrate that sexual stimuli capture the attentional resources of individuals with PIPU during the early automatic stages of attentional processing, whereas there appears to be attentional avoidance of sexual stimuli during the late conscious stages of attentional processing.

Collectively, the available evidence seems to suggest that individuals with PIPU may exhibit biased attentional processing in two ways: (a) enhanced attentional engagement (i.e., automatic orientation to sexual stimuli) and (b) enhanced attentional disengagement (i.e., strategic redirection of attention away from sexual stimuli). Unfortunately, no studies have examined the nature of sex-related attentional bias in PIPU. The assessment instruments mentioned in the above studies do not allow a direct distinction between attentional engagement with a cue and attentional disengagement from this cue. These instruments tend to yield an overall indicator of attentional bias, in which engagement and disengagement processes are intertwined.

To examine the attentional components involved in PIPU, the present study employed an exogenous cueing task (32) that can simultaneously examine three attentional processing modes: enhanced attentional engagement, delayed attentional disengagement, and enhanced attentional disengagement (i.e., attentional avoidance). In this task, a cue appears in one of two locations. On most trials (the congruent cue condition), a target appeared at the cued position, and on the remaining trials (the incongruent cue condition), a target appeared at the alternative position. The acceleration on congruently cued trials has resulted from the benefit of attentional engagement with the cued location. The slowdown on incongruently cued trials has been related to the cost of having to disengage attention from the cued location. Because attentional allocation patterns can change over time (33), this study uses two stimulus presentation times (100 and 500 ms) to differentiate between the early and later stages of attention processing. Engagement is believed to occur early in the attentional processing of addictive stimuli, whereas disengagement reflects attempts to cope at a later stage (34).

The following hypotheses were tested in the present study: (1) participants with high PIPU tendencies would show an early (i.e., 100 ms) enhanced engagement with pornographic images compared with participants with low PIPU tendencies; (2) participants with high PIPU tendencies would subsequently (i.e., 500 ms) show greater attentional disengagement from pornographic images than those with low PIPU tendencies; and (3) the extent of both engagement and disengagement would correlate with the severity of PIPU symptoms.

## Methods

### Participants

Eighty male college students (*M*_age_ = 19.69, *SD* = 1.77) were recruited from a pool of 273 male college students who had previously completed the Problematic Internet Pornography Use Scale [PIPUS; (35); Chinese translation by Chen and Jiang (36)]. Given that the literature consistently shows that PIPU is more prevalent in men than in women [e.g., (2, 37–39)], female participants were excluded from this study. Because PIPU is not a coded diagnosis and there are no uniform diagnostic criteria, participants are selected according to the highest and lowest quartiles of their PIPUS scores (13). Based on this categorization criterion, 80 participants (40 participants were included in the high PIPU tendency group, and 40 were included in the low PIPU tendency group) were invited to voluntarily participate in the follow-up behavioral experiment. All participants were heterosexual. The main exclusion criteria were being younger than 18 years, having a history of substance abuse, and using psychotropic medication.

### Measurement instruments and procedure

Problematic Internet pornography use was assessed using the Chinese version of PIPUS (36). The scale consists of 12 items with four dimensions: distress and functional problems, overuse, difficulty with self-control, and use to escape negative emotions. Using a six-point Likert-type scale (0 for “never,” 5 for “all the time”), participants were asked to report Internet pornography use over the past 6 months. Problematic Internet Pornography Use Scale has been shown to have good reliability and validity (19, 40). The Cronbach's α in the present study is 0.95.

The participants first completed the PIPUS. Subsequently, 80 participants, screened according to the above criteria, completed the exogenous cueing task. After completing the behavioral task, the participants completed the Self-Rated Depression Scale [(41); Cronbach's α = 0.80] and Self-Rated Anxiety Scale [(42); Cronbach's α = 0.86] to measure their depression and anxiety levels. Finally, they were debriefed and compensated for their time.

### Stimuli and experimental task

The stimuli consisted of 48 color pictures selected from free websites. Of these, 24 were pornographic stimuli depicting four different types of heterosexual sex (vaginal, anal, cunnilingus, and oral sex), and 24 were neutral images depicting a man and woman walking or jogging. Pornographic and neutral images were matched for the number and sex of the individuals. These images were used in our previous study [see (19)]. Each image was 6 cm high and 6 cm wide on a computer monitor. These pictures served as cues in the experimental task. The target stimulus to which participants needed to respond was a black square with a side length of 0.5 cm. Cue and target stimuli were displayed in two gray square boxes with 6 cm sides, 3.5 cm to the left and right of the central fixation point. The stimuli were presented using E-Prime 2.0.

**Figure 1:**
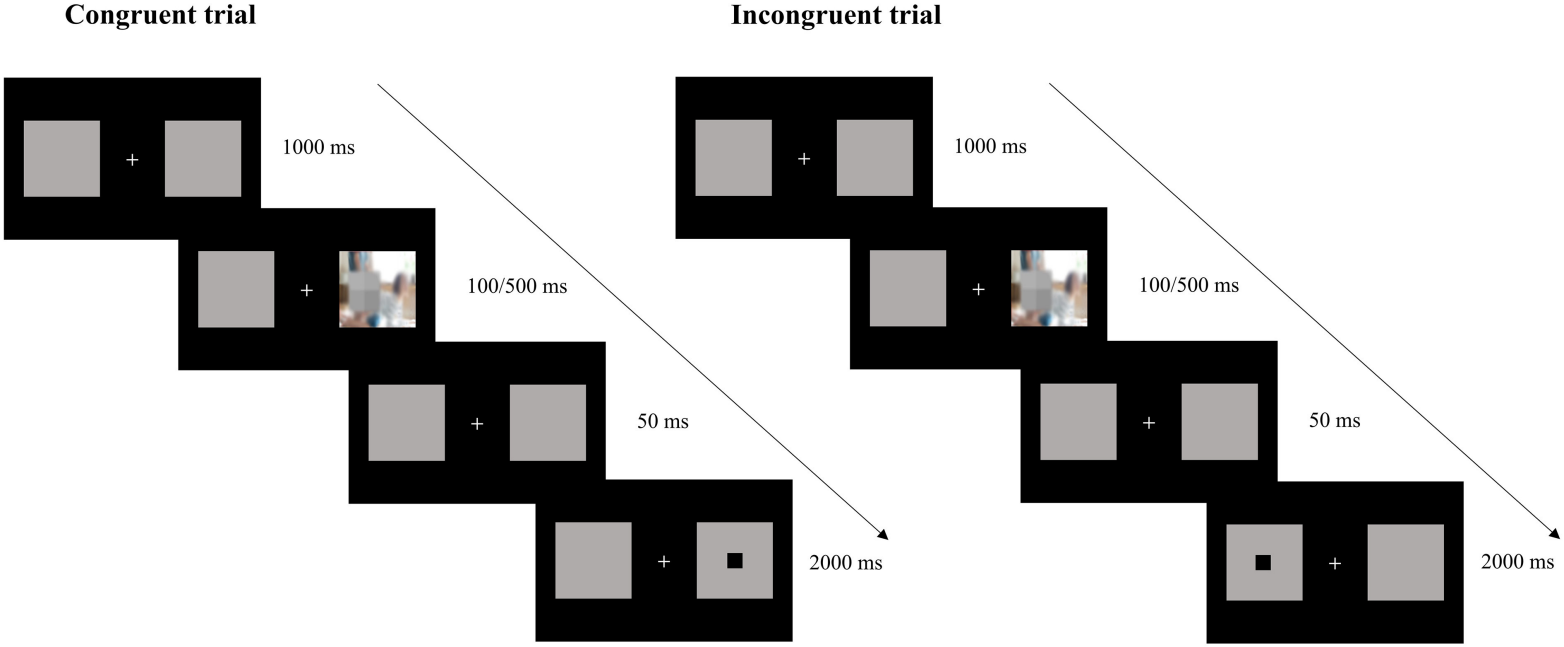
Procedure of the exogenous cueing task. The two columns illustrate a congruent and an incongruent trial, respectively.

The participants sat approximately 60 cm in front of a monitor. Each trial started with a central fixation point flanked by two empty squares and was presented for 1,000 ms. Subsequently, a picture cue was displayed in one of the boxes for 100 or 500 ms. Then, the cue disappeared, and after 50 ms, the target (a black square) appeared in the left or right box. Participants were tasked to determine the location of the target as quickly and accurately as possible, pressing the “1” key on a standard computer keyboard when the target appeared on the left and the “2” key when the target appeared on the right. The target disappeared after the key was pressed, and the longest presentation time was 2,000 ms (Figure 1).

The task consisted of 12 practice trials and 192 experimental trials (divided into two blocks of 96 trials). Two-thirds of the experimental trials (128 trials, 64 pornographic and 64 neutral) were congruent (i.e., the target and cue appeared in the same position), and one-third (64 trials, 32 pornographic and 32 neutral) were incongruent (i.e., the target and cue appeared in the opposite position). Each picture was presented four times, as congruent and incongruent trials, for both 100 and 500 ms. Picture cues and targets appeared with the same probability on the left or right box. All trials were presented in random order.

### Data reduction

Incorrect responses (1.23% of trials) were excluded from analysis. In line with previous studies (43), reaction times (RTs) lower than 150 ms or higher than 1,200 ms were considered outliers. Moreover, RTs deviating by more than three SDs from the individual mean RT were deleted. Normal distribution of the data was demonstrated by the Shapiro–Wilk test. Two types of attentional bias were calculated for short- and long-picture time trials. Attentional engagement was calculated based on congruent trials by subtracting the RTs for pornographic stimuli from those for neutral stimuli. A positive attentional engagement score reflects that attention is grabbed by pornographic stimuli more than by neutral stimuli. Attentional disengagement was calculated based on incongruent trials by subtracting the RTs for neutral stimuli from those for pornographic stimuli. A positive attentional disengagement score indicates greater difficulty in disengagement from pornographic stimuli than from neutral stimuli. Conversely, negative scores for engagement and disengagement bias suggest avoidance biases, namely, a tendency to pay attention away from pornographic stimuli.

### Statistical analysis

Group differences in age, PIPUS score, depression, and anxiety were analyzed using an independent sample *t*-test. To examine the differences between individuals with high and low PIPU tendencies in attentional bias for pornographic stimuli, a 2 × 2 × 2 × 2 mixed-design analysis of variance (ANOVA) was performed, with Stimulus Type (pornography/neutral), Congruency (congruent/incongruent), and Stimulus Duration (100/500 ms) as within-subjects variables, and Group (high/low PIPU) as the between-subjects variable. A one-sample *t*-test was used for attentional engagement and disengagement to test whether they were significantly different from zero. Partial correlation analysis (controlling for RTs to neutral cues) was used to examine the relationship between attentional bias and PIPU scores.

### Ethics

This study was conducted in accordance with the Declaration of Helsinki, and approved by the local ethics committee. The participants provided written informed consent after they understood the contents of the experiment.

## Results

### Group characteristics

Group characteristics are presented in Table 1. As expected, individuals with high tendencies toward PIPU had higher PIPUS scores than those with low tendencies toward PIPU, *t*_(78)_ = 14.06, *p* < 0.001, Cohen's *d* = 3.14. Moreover, the high PIPU tendency group was more depressed and anxious than the low PIPU tendency group, as indicated by the SDS, *t*_(78)_ = 2.74, *p* = 0.008, Cohen's *d* = 0.61, and SAS scores, *t*_(78)_ = 2.30, *p* = 0.024, Cohen's *d* = 0.51, respectively.

**Table 1:** Group characteristics of the study sample.

**Variable (M ±SD)**	**High tendencies toward PIPU (*n* = 40)**	**Low tendencies toward PIPU (*n* = 40)**	***t***	***p***	***d***
Age (years)	19.80 ± 1.83	19.58 ± 1.72	0.57	0.573	0.13
PIPUS	26.43 ± 11.28	1.28 ± 0.78	14.06	<0.001	3.14
SDS	35.18 ± 2.99	33.10 ± 3.75	2.74	0.008	0.61
SAS	34.05 ± 3.33	32.28 ± 3.57	2.30	0.024	0.51

PIPUS, Problematic Internet Pornography Use Scale; SDS, Self-Rated Depression Scale; SAS, Self-Rated Anxiety Scale.

### Descriptive statistics

**Figure 2:**
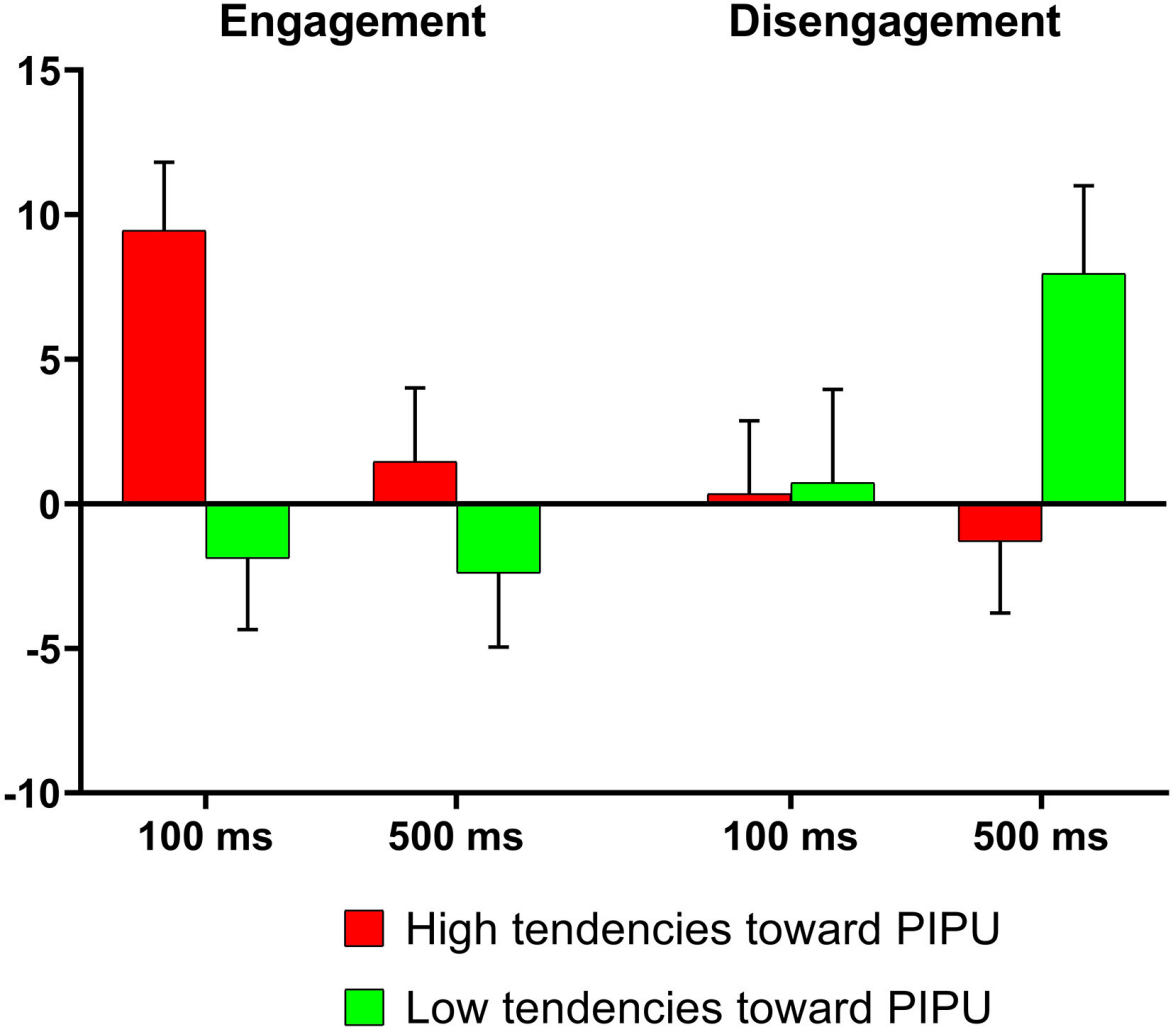
Attentional bias scores as a function of group (PIPU, problematic Internet pornography use).

The mean and SD of the RTs based on group and trial are shown in Table 2. The attentional bias indices calculated from these RTs are shown in Figure 2. Within each group, a one-sample *t*-test was conducted for each attentional bias index to test whether these indices were significantly different from zero. Individuals with high tendencies toward PIPU exhibited significantly stronger attentional engagement with pornographic pictures than with neutral pictures in the short image time trials, *t*_(39)_ = 4.00, *p* < 0.001, Cohen's *d* = 0.63. Furthermore, individuals with low tendencies toward PIPU showed impaired disengagement from pornographic pictures than from neutral pictures in the long image time trials, *t*_(39)_ = 2.61, *p* = 0.013, Cohen's *d* = 0.41. No other attentional bias indices were significantly different from zero.

**Table 2:** Mean and SD of the RTs in the exogenous cueing task on the basis of group and trial.

**Duration**	**Congruency**	**Stimulus type**	**High tendencies toward PIPU (*n* = 40)**	**Low tendencies toward PIPU (*n* = 40)**
			**M ±SD**	**M ±SD**
100 ms	Congruent	Pornographic	347.06 ± 43.80	353.02 ± 41.06
		Neutral	356.51 ± 50.53	351.13 ± 42.40
	Incongruent	Pornographic	406.72 ± 52.71	401.24 ± 42.86
		Neutral	406.39 ± 50.85	400.51 ± 45.99
500 ms	Congruent	Pornographic	360.67 ± 46.93	362.66 ± 50.28
		Neutral	362.12 ± 47.13	360.26 ± 52.22
	Incongruent	Pornographic	403.34 ± 60.18	393.79 ± 51.25
		Neutral	404.65 ± 58.31	385.84 ± 44.98

PIPU, problematic Internet pornography use.

After controlling for RTs to neutral cues, partial correlations between the attentional bias indicators and PIPUS scores were performed. The results showed that PIPU symptoms were positively correlated with attentional engagement scores in the short picture time trials (*r* = 0.36, *p* = 0.001) and weakly negatively correlated with attentional disengagement in the long picture time trials (*r* = −0.22, *p* = 0.054).

### Group difference analysis on attentional bias indices

A 2 (Group) × 2 (Stimulus Valence) × 2 (Congruency) × 2 (Stimulus Duration) repeated-measures ANOVA on RTs revealed a significant main effect of congruency, with faster responses to the congruent cues (*M* = 356.68 ms, *SD* = 41.23) than incongruent cues (*M* = 400.31 ms, *SD* = 47.63), *F*_(1,78)_ = 297.34, *p* < 0.001, $\;\eta {\;}_{p}^{2}$ = 0.79. Furthermore, group × stimulus valence, *F*_(1,78)_ = 5.30, *p* = 0.024, $\;\eta {\;}_{p}^{2}$ = 0.06, group × congruency, *F*_(1,78)_ = 5.18, *p* = 0.026, $\;\eta {\;}_{p}^{2}$ = 0.06, stimulus valence × congruency, *F*_(1,78)_ = 6.54, *p* = 0.012, $\;\eta {\;}_{p}^{2}$ = 0.08, and congruency × stimulus duration, *F*_(1,78)_ = 14.47, *p* < 0.001, $\;\eta {\;}_{p}^{2}$ = 0.16, were also significant. Importantly, the crucial Group × Stimulus Valence × Congruency × Stimulus Duration interaction was significant, *F*_(1,78)_ = 9.17, *p* = 0.003, $\;\eta {\;}_{p}^{2}$ = 0.11. To clarity the four-way interaction further, we performed separate ANOVAs for each stimulus duration.

#### 100 ms condition

At this stimulus duration, a Group × Stimulus Valence × Congruency interaction was found, *F*_(1,78)_ = 4.33, *p* = 0.041, $\;\eta {\;}_{p}^{2}$ = 0.05. In order to simplify analyses, separate ANOVAs were performed for each group. For the high tendencies toward PIPU group, the Stimulus Valence × Congruency interaction was significant, *F*_(1,39)_ = 7.75, *p* = 0.008, $\;\eta {\;}_{p}^{2}$ = 0.17. The simple-effect analyses of the two-way interaction revealed faster RTs in congruent trials after pornography cues (*M* = 347.06 ms, *SD* = 43.80) than after neutral cues (*M* = 356.51 ms, *SD* = 50.53), *F*_(1,39)_ = 15.98, *p* < 0.001, $\;\eta {\;}_{p}^{2}$ = 0.29, thus exhibiting a significantly enhanced attentional engagement with pornography pictures (see Figure 2). In the incongruent trials, the RTs for pornography cues (*M* = 406.72 ms, *SD* = 52.71) did not differ for neutral cues (*M* = 406.39 ms, *SD* = 50.85), *F*_(1,39)_ = 0.02, *p* > 0.89. For the low tendencies toward PIPU group, the Stimulus Valence × Congruency interaction was not significant, *F*_(1,39)_ = 0.09, *p* > 0.76.

#### 500 ms condition

Again, a Group × Stimulus Valence × Congruency interaction was found, *F*_(1,78)_ = 4.20, *p* = 0.044, $\;\eta {\;}_{p}^{2}$ = 0.05. We performed separate ANOVAs for each group. For the high tendencies toward PIPU group, the Stimulus Valence × Congruency interaction was not significant, *F*_(1,39)_ = 0.00, *p* > 0.96. For the low tendencies toward PIPU group, the Stimulus Valence × Congruency interaction was significant, *F*_(1,39)_ = 7.13, *p* = 0.011, $\;\eta {\;}_{p}^{2}$ = 0.16. The simple-effect analyses revealed slower RTs in incongruent trials after pornography cues (*M* = 393.79 ms, *SD* = 51.25) than after neutral cues (*M* = 385.84 ms, *SD* = 44.98), *F*_(1,39)_ = 6.83, *p* = 0.013, $\;\eta {\;}_{p}^{2}$ = 0.15, thus exhibiting a significantly enhanced attentional disengagement with pornography pictures. In the congruent trials, the RTs for pornography cues (*M* = 362.66 ms, *SD* = 50.27) did not differ for neutral cues (*M* = 360.26 ms, *SD* = 52.22), *F*_(1,39)_ = 0.45, *p* > 0.50.

## Discussion

The primary objective of the present study was to investigate the differences between individuals with high and low PIPU tendencies in their attentional bias to sex-related and neutral images over time during an exogenous cueing task. To examine the nature of sex-related attentional bias in PIPU, attentional engagement and disengagement scores were measured over time. These attentional indices revealed that individuals with high PIPU tendencies were significantly more vigilant for pornographic images than for neutral images, relative to individuals with low tendencies toward PIPU at first (100 ms). However, over time (500 ms), individuals with high tendencies toward PIPU were significantly less attentive to pornographic images than those with low tendencies toward PIPU. Moreover, partial correlation analysis showed that the severity of PIPU symptoms was positively correlated with attentional engagement scores in the short picture-time trials (100 ms) and weakly negatively correlated with attentional disengagement scores in the long picture-time trials (500 ms). To our knowledge, this is the first study to distinguish between attentional engagement and disengagement when examining attentional bias toward sexual cues in the context of PIPU.

The findings of this study suggest that the attentional bias mechanism that underlies PIPU may not be a simultaneous but a sequential process (i.e., following a pattern from vigilance to avoidance). This finding agrees with previous results that individuals with PIPU exhibit a stronger attentional bias to sexual cues relative to healthy controls when the stimulus presentation time is 150–220 ms (13, 29), whereas group differences disappear when the presentation time is greater than 500 ms (30, 31). According to the Reinforcement/Reprocessing model of Reflectivity (44), conditioning processes and learned associations have different intensity levels, and different intensity levels produce different activation speeds. Stronger associations occur faster than weaker associations. Attentional engagement with sex-related stimuli is shaped by repeated pornography consumption that is linked to positive implications [e.g., sexual arousal; (22)]. Therefore, attentional engagement reflects early, rapid, and automatic processes. On the other hand, attentional avoidance of sex-related stimuli is shaped by the negative consequences of consumption (e.g., anticipation of loss of control). As attentional avoidance involves weighing the pros and cons of pornography consumption, it reflects a late, slow, and conscious process. Because pornography consumption may be more strongly associated with positive outcomes than with negative outcomes, individuals with PIPU rapidly detect sexual cues and subsequently avoid them (44).

The presence of an attentional bias to sexual stimuli presented for 100 ms, followed by the absence of attentional bias at 500 ms in individuals with high PIPU tendencies, is also compatible with the vigilance–avoidance pattern of visual attention for addiction-related stimuli in substance use disorder [see (45)]. For example, Noël et al. (46) found that abstinent alcoholics exhibited enhanced attentional bias for brief (50 ms) alcohol-related pictures. However, this bias toward alcohol cues disappeared when stimulus presentation times were extended to 500 and 1,250 ms. In their view, the lack of attentional bias to alcohol-related pictures reflects abstinent alcoholics' efforts to remain abstinent (46). Similarly, Lee et al. (47) used eye-tracking techniques to find that problematic drinkers with ambivalence had a tendency to direct their gaze toward alcohol cues at the onset of the trial but away from alcohol cues during the final phase of the trial. In addition, closely related to the findings of this study, an approach–avoidance tendency (i.e., either approaching or inhibiting pornography use) was found in PIPU. For example, Schiebener et al. (48) used an executive multitasking paradigm with two sets of pornographic and neutral images. Participants were asked to perform all classification tasks in equal amounts, although they were free to switch between sets and tasks. It was found that individuals with cybersex addiction either overused (approach) or neglected (avoidance) pornographic images. Because the multitasking task was not specifically designed to assess approach or avoidance, Snagowski and Brand (49) conducted another study using an approach–avoidance task in which participants used a joystick to either push pornographic pictures away or pull them toward themselves. They found that the relationship between approach/avoidance tendencies and cybersex addiction was not linear but quadratic. That is, individuals with high cybersex addiction tended to either show approach or avoidance tendencies to pornographic pictures.

Recently, Field et al. (27) proposed a new theoretical explanation for attentional bias in obesity and addiction, emphasizing that appetitive and aversive motivational processes jointly determine attentional bias. Both addiction and obesity are characterized by motivational conflict or ambivalence. Addictive stimuli are both desired by addicts but also threaten their goals of changing behavior (50). Attentional bias is derived from current evaluations of substance-related cues, which are likely to change over time, depending on the current motivational orientation for substance consumption or restriction. Consequently, the observed approach–avoidance pattern of attentional bias is related to motivational conflict (27). According to this theory, individuals with high PIPU may experience motivational conflicts or ambivalence. On the one hand, they are attracted to pornographic stimuli; on the other hand, pornographic stimuli can trigger concerns about problem behaviors, causing them to avoid pornographic stimuli to regulate their negative emotions or suppress their subjective cravings (48, 49).

Considering the role of motivational conflict may explain the inconsistent results of previous attentional bias studies in the field of PIPU. Previous studies using the dot-probe task found that individuals with PIPU exhibited a stronger attentional bias to sexual cues relative to healthy controls when the stimulus presentation time was 150 ms (29), whereas group differences disappeared when the presentation time was 500 ms (30). However, studies using the addiction Stroop task found that when the stimulus presentation time was 500 ms, individuals with PIPU were still found to have an attentional bias toward sexual cues (13, 28). An important reason is that Stroop interference cannot be unambiguously interpreted as attentional bias, it may also arise from individuals' attempts to suppress their attentional bias (51). Identical patterns of Stroop interference are produced by appetitive and aversive cues. According to the model proposed by Field et al. (27), when using the addiction Stroop task to assess attentional bias, the magnitude of the attentional bias depends on the overall evaluation strength of addiction-related cues, rather than its valence (positive, negative, or ambivalence). However, when the dot-probe task was employed, positive evaluations led to attentional bias toward addition-related cues, while ambivalent evaluations led to an approach–avoidance pattern of attentional bias.

This study has several important theoretical and clinical implications. First, CSBD is currently classified as an impulse control disorder in the ICD-11. However, many scholars have suggested that it should be considered a behavioral addiction [e.g., (8, 52)]. The findings of this study suggest that, with respect to approach–avoidance pattern of attentional bias, PIPU is similar to substance use disorder [e.g., abstinent alcoholics; (46)]. The approach-avoidance framework is also consistent with the dual-process model of addiction [e.g., (17)], which argues that automatic and controlled processes play an important role in the development and maintenance of addiction disorders. However, further research is needed to confirm whether individuals who experience motivational conflict about pornographic stimuli have different patterns of attentional bias compared to those who do not. Gambling disorder has been reclassified from impulse control disorders to addiction disorders in DSM-5 and ICD-11. Additional studies are needed to determine the most appropriate classification for PIPU (and more generally CSBD). Second, it is important to distinguish between attentional engagement and disengagement. Previous studies on attentional bias in the PIPU domain have reported mixed results; it is likely that the adopted tasks yield an overall index of attentional bias without distinguishing between initial attention and the difficulty of disengagement. Third, it is important to consider the timing of the stimulus presentation. There seems to be a time difference between the activation of approach and avoidance tendencies, and the approach–avoidance pattern of attentional bias occurs sequentially. Finally, in a clinical setting, the vigilance–avoidance pattern exhibited by patients with PIPU may be a behavioral marker of the presence of motivational conflict or ambivalence. This is an important opportunity for treatment because patients seek change. At this point, the therapist can attempt to weaken the association between sexual stimuli and positive outcomes while reinforcing the association between sexual stimuli and adverse outcomes. For example, previous research has found that when attention is repeatedly diverted from a stimulus, the value of that stimulus decreases (53). Therefore, one intervention is to use attentional bias modification to alter the evaluation of pornographic cues. This, in turn, may reduce the ability of this cue to elicit attentional bias, craving and motivated behavior. Furthermore, evaluative conditioning procedures can be employed to pair sexual-related cues and negative stimuli, which may alter the valence of sexual-related cues [e.g., (54)]. Using the above methods to change the pattern of approach/avoidance of attentional bias may be beneficial to reduce and control pornography consumption in individuals with PIPU. Further, previous studies have demonstrated that the dopaminergic system, with projections from the ventral tegmental area to the striatum, the anterior cingulate cortex, and other prefrontal brain regions, is involved in the occurrence of attentional bias (23, 55). These cortical and subcortical brain regions are also main correlates of substance addiction and other behavioral addictions (52). Future intervention research employing attentional retraining may gain valuable information by identifying those patients with higher levels of brain activity to addiction-related cues, especially in prefrontal cortex regions.

This study had several limitations. First, the sample for this study consisted of college students who were identified using a self-reported scale. Future studies should replicate the results of this study by using clinical samples. Second, the RT indicators derived from the exogenous cueing task in this study provided an indirect measure of attentional bias. Further studies should employ more direct measures of attentional bias [e.g., eye-tracking technology; see (56)]. Third, we speculated that individuals with high PIPU have ambivalence toward pornographic stimuli; however, this was not measured in this study. Therefore, an important task for future studies is to measure ambivalence scores in porn addicts and examine differences in attention patterns between porn addicts with and without ambivalence (47). Fourth, the study design could not establish a causal relationship between PIPU and attentional bias. Further studies are required to clarify the direction of this relationship. For instance, researchers could employ an attentional bias modification task [e.g., (57)] to modify the attentional engagement or disengagement and examine whether this modification affects an individual's PIPU symptoms. Fifth, only pornographic and neutral pictures were included in this study. Further research should add an additional category of pictures (e.g., another type of reward) to confirm whether attentional bias occurs for the pornographic stimuli in particular or the arousing stimuli in general. Finally, only male participants were investigated in this study because the prevalence of PIPU is higher in males than in females, and males appear to be more likely to encounter problems. Future research should examine whether a similar pattern of attentional biases exists in women.

## Conclusions

This is the first study to examine attentional bias toward sex-related stimuli between individuals with high and low PIPU tendencies with an exogenous cueing task designed to differentiate between attentional engagement and disengagement. This study provides the first evidence of an approach–avoidance pattern of attentional bias toward pornographic stimuli in individuals with high PIPU tendencies. More specifically, individuals with high tendencies toward PIPU exhibited an automatic attentional preference for sex-related stimuli with high incentive salience, followed by attentional avoidance at the late stage of attentional processing, which reflects a strategic process of reducing subjective discomfort. In addition, our data showed that the approach–avoidance pattern of attentional bias was significantly related to the severity of PIPU symptoms.

## Data availability statement

The raw data supporting the conclusions of this article will be made available by the authors, without undue reservation.

## Ethics statement

The studies involving human participants were reviewed and approved by Chengdu Medical College. The patients/participants provided their written informed consent to participate in this study.

## Author contributions

JW and YH contributed to the conceptualization and supervision. JW contributed to the formal analysis and writing—original draft. YH contributed to the writing—review and editing.

## Funding

This work was supported by grants from the Sichuan Sex Sociology and Sex Education Research Center (SXJYB2212), the Disciplinary Construction Innovation Team Foundation of Chengdu Medical College (CMC-XK-2105), Sichuan Social Sciences Planning Fund Program (SC20EZD010), and the Foundation of Sichuan Psychological Society (SCSXLXH2021042).

## Conflict of interest

The authors declare that the research was conducted in the absence of any commercial or financial relationships that could be construed as a potential conflict of interest.

## Publisher's note

All claims expressed in this article are solely those of the authors and do not necessarily represent those of their affiliated organizations, or those of the publisher, the editors and the reviewers. Any product that may be evaluated in this article, or claim that may be made by its manufacturer, is not guaranteed or endorsed by the publisher.
